# Exercise–microbiota interactions in aging‐related sarcopenia

**DOI:** 10.1002/jcsm.12942

**Published:** 2022-02-10

**Authors:** Johannes Burtscher, Andrea Ticinesi, Gregoire P. Millet, Martin Burtscher, Barbara Strasser

**Affiliations:** ^1^ Institute of Sport Sciences University of Lausanne Lausanne Switzerland; ^2^ Department of Biomedical Sciences University of Lausanne Lausanne Switzerland; ^3^ Department of Medicine and Surgery University of Parma Parma Italy; ^4^ Microbiome Research Hub (MRH) University of Parma Parma Italy; ^5^ Geriatric‐Rehabilitation Department Parma University‐Hospital Parma Italy; ^6^ Department of Sport Science University of Innsbruck Innsbruck Austria; ^7^ Medical Faculty Sigmund Freud Private University Vienna Austria; ^8^ JPI‐HDHL Knowledge Platform on Food, Diet, Intestinal Microbiomics and Human Health The Netherlands Organisation for Health Research and Development Amsterdam The Netherlands

Sarcopenia, the age‐related loss of skeletal muscle mass and function, is associated with increasing burden of frailty, disability, and mortality for our aging society. Nevertheless, the underlying cellular and molecular mechanisms and the role of life‐style factors are insufficiently understood. Exercise is one such factor and recent evidence supports the potential of strength training alone or combined with aerobic exercise to mitigate sarcopenia.^1^ Gut microbiota are implicated in the development of muscle loss during aging as well, as recently systematically reviewed in this journal.^2^ Consequently, the combination of appropriate exercise programmes and dietary interventions aimed at modifying gut microbiota hold great promise to counteract sarcopenia. However, the evaluation of such combined approaches led to ambiguous results, with one recent meta‐analysis supporting favourable effects on aging‐related sarcopenia^3^ and another not.^4^ Herein, we discuss potential reasons for those discrepancies, elucidate the complex interactions between exercise, gut microbiota and skeletal muscle health, and suggest appropriate intervention strategies to prevent aging‐related sarcopenia.

## Type and dose of exercise in the prevention of aging‐related sarcopenia

Various exercise programmes, including single or combined exercise types, for example, aerobic and/or resistance exercise, have been demonstrated to reduce aging‐related sarcopenia in healthy and diseased people.^5^, ^6^ In obese individuals aged >64 years old, weight loss combined with both aerobic and resistance training (RT) most efficiently preserved lean mass, physical function, and reduced frailty.^7^ This combination is likely the most promising strategy to maintain and/or improve muscle mass and strength.^1^

Evidence‐based guidelines recommend the adoption of either a combination of RT and power training or high‐intensity interval training (HIIT) against age‐related sarcopenia.^8^, ^9^, ^10^ Moderate to high (60% to 80% of 1‐RM) intensities are optimal for RT, while low to moderate (e.g. lighter loading and high movement velocity) intensities are preferable for power training to stimulate the speed component (≤60% of 1‐RM) for adults that are already physically strong as a prerequisite for power performance.^11^ The volume of RT should be adapted to preexisting muscular fitness: from 3 to 6 sets per muscle group per week (for beginners) to a maximum of 10 sets per muscle group per week (for advanced) of 10–15 repetitions per set. Conversely, 85% to 95% of the maximum heart rate should be the target for HIIT, which should be performed for 4 × 4′ intervals or at intensities greater than peak aerobic capacity for 5 × 1′ intervals to improve muscle mass and physical function in a short time frame.^10^ Exercise schedules need to be adapted to the individual performance status. Risks for power training include exaggerated exhausting, especially for sedentary older people, increased blood pressure, and during the first weeks of training, joint limitations, and pain. Regarding HIIT, untrained older people may be unable to adhere to the target heart rate. Despite the potential of exercise to counteract sarcopenia, poor adherence and lack of sufficient physical fitness may indeed represent the main barriers for the implementation of exercise programmes in older people.^2^

## Effects of exercise on gut microbiota

Growing evidence suggests that physical activity (including exercise) can trigger favourable changes in the qualitative and quantitative gut microbial composition and metabolic function, resulting in health benefits for the host.^12^ These changes are independent of diet and may depend on type and intensity of exercise.^13^

Athletes generally exhibit higher biodiversity and representation of bacterial taxa with anti‐inflammatory properties and capacity to synthetize short‐chain fatty acids (SCFAs) in their faecal microbiota than sedentary controls.^14^, ^15^ Compared with community‐dwelling older adults, master athletes display a more homogeneous composition of gut microbiota, which was associated with positive health benefits, such as psychological well‐being, most likely due to changes in the gut–brain axis.^16^

Both aerobic training and RT also showed significant modifications of faecal microbiota composition after the implementation of an exercise programme in both younger and older individuals.^17^, ^18^ These modifications included an increased representation of *Bifidobacteria* and *Faecalibacterium prausnitzii* and were associated with higher stool levels of butyrate.^17^, ^18^ *Bifidobacteria* can positively modulate the host immunity through the up‐regulation of anti‐inflammatory cytokines, and T cell regulation, while SCFAs, and particularly butyrate, a microbial metabolite synthetized by *F. prausnitzii* among others, is a well‐known regulator of the host metabolic balance.^19^, ^20^ Interestingly, such changes were influenced by the pre‐existing obesity status, but were independent of diet, and rapidly disappeared after the exercise intervention.^17^

The effects of exercise programmes on the aging gut microbiota are less clear, because in older individuals overweight, chronic inflammatory states, multimorbidity, and polypharmacy progressively can promote gut microbiota dysbiosis with increased representation of opportunistic pathogens.^21^, ^22^ In addition, exercise‐induced microbiota alterations seem to be more substantial in earlier life compared with later life.^23^ However, recent findings from the American Gut Project revealed that chronic exercise benefits especially overweight elderly individuals by maintaining gut microbiota stability (composition and function).^24^ Importantly, excessive exercise, for example, disproportionate to training levels, or exercise in hot environments, can induce unfavourable changes in gut microbiota composition and disrupt the gut mucosal barrier, resulting in a paradox pro‐inflammatory effect for the host.^25^, ^26^

## Effects of gut microbiota on skeletal muscle and aging‐related sarcopenia

Several in vitro experiments and preclinical and clinical studies provide direct and indirect evidence for the interplay between gut microbiota and muscle mass.^27^, ^28^ Age‐related decline in muscle mass and function was suggested to be associated with a distinct gut microbiota composition towards dysbiosis.^29^, ^30^ The composition of gut microbiota has further been linked to obesity and various metabolic diseases, including type 2 diabetes.^31^, ^32^

The emerging concept of the gut‐muscle axis assumes a reciprocal effect between these organs. While the mechanistic underpinnings of gut and muscles interactions are still poorly understood, the influence of gut microbiota on the general regulation of the host metabolism is well established and a promising research field. The synthesis of SCFAs by gut microbiota, resulting from microbial metabolism of ingested plant fibres, is thought to be favourable for the host metabolism, including via increased insulin sensitivity, muscle anabolism and modulation of age‐related chronic inflammation.^19^ These effects are particularly pronounced for butyrate and have a relevant influence on fuel availability and exercise capacity.^33^ On the other hand, gut microbiota dysbiosis resulting from sedentary lifestyle and unhealthy dietary patterns can be associated with increased intestinal mucosa permeability and absorption of bacterial metabolites and endotoxins that promote low‐grade systemic inflammation and insulin resistance.^15^, ^31^, ^32^

Animal experimentations indicate a bidirectional communication between gut and skeletal muscle and point out that gut microbiota is critical for optimal muscle function.^34^, ^35^ In fact, the presence of an intact gut microbiome is necessary for normal muscle adaptations to exercise^36^ and to promote adequate dietary protein digestion and amino acid absorption, a critical processes to counteract sarcopenia‐associated muscle protein wasting.^37^, ^38^

## A putative role of mitochondria in exercise–microbiota–muscle interactions

Mitochondrial dysfunction has emerged as a central factor in the pathogenesis of age‐related sarcopenia.^39^ While the integral role of mitochondrial deficits in muscle degeneration^39^ as well as the benefits of various types of exercise on skeletal muscle mitochondria^40^ are widely accepted, it is still poorly understood whether exercise‐induced benefits on the muscle‐gut axis are also partially mediated via mitochondria.

Among the numerous direct effects of regular exercise on muscle mitochondria are improved energy metabolism, mitochondrial biogenesis, as well as antioxidative and immune capacities.^40^ However, exercise also affects mitochondria in tissues remote from skeletal muscle, such as the brain^41^ and possibly the gut.^42^ How exercise‐induced muscle mitochondria benefits are communicated to (mitochondria in) other tissues is a topic of intense investigation and involves signalling via myo/mitokines, micro‐RNAs, and metabolites.^41^

On the other hand, gut microbiota are increasingly recognized to also exert direct and indirect effects on mitochondria,^43^ in particular during exercise. Mediators of these interactions may be SCFAs and secondary bile acids, but also gut hormones and redox or inflammatory signalling. A recent study demonstrated that germ‐free mice had atrophic skeletal muscles with impaired mitochondrial functions.^44^ Transplantation of gut microbiota in these mice increased both skeletal muscle mass and mitochondrial function, supporting an important role of gut microbiota on skeletal muscle mitochondria. In horses, it was shown that specifically butyrate‐producing bacteria of the gut microbiome were involved in modulating mitochondria‐related gene expression, possibly impacting energy metabolism, oxidative stress, and inflammation.^45^ Conversely, mitochondria may also modulate gut microbiota, via mechanisms including redox signalling, immune system activation and intestinal barrier function modulation.^42^

**Figure 1:**
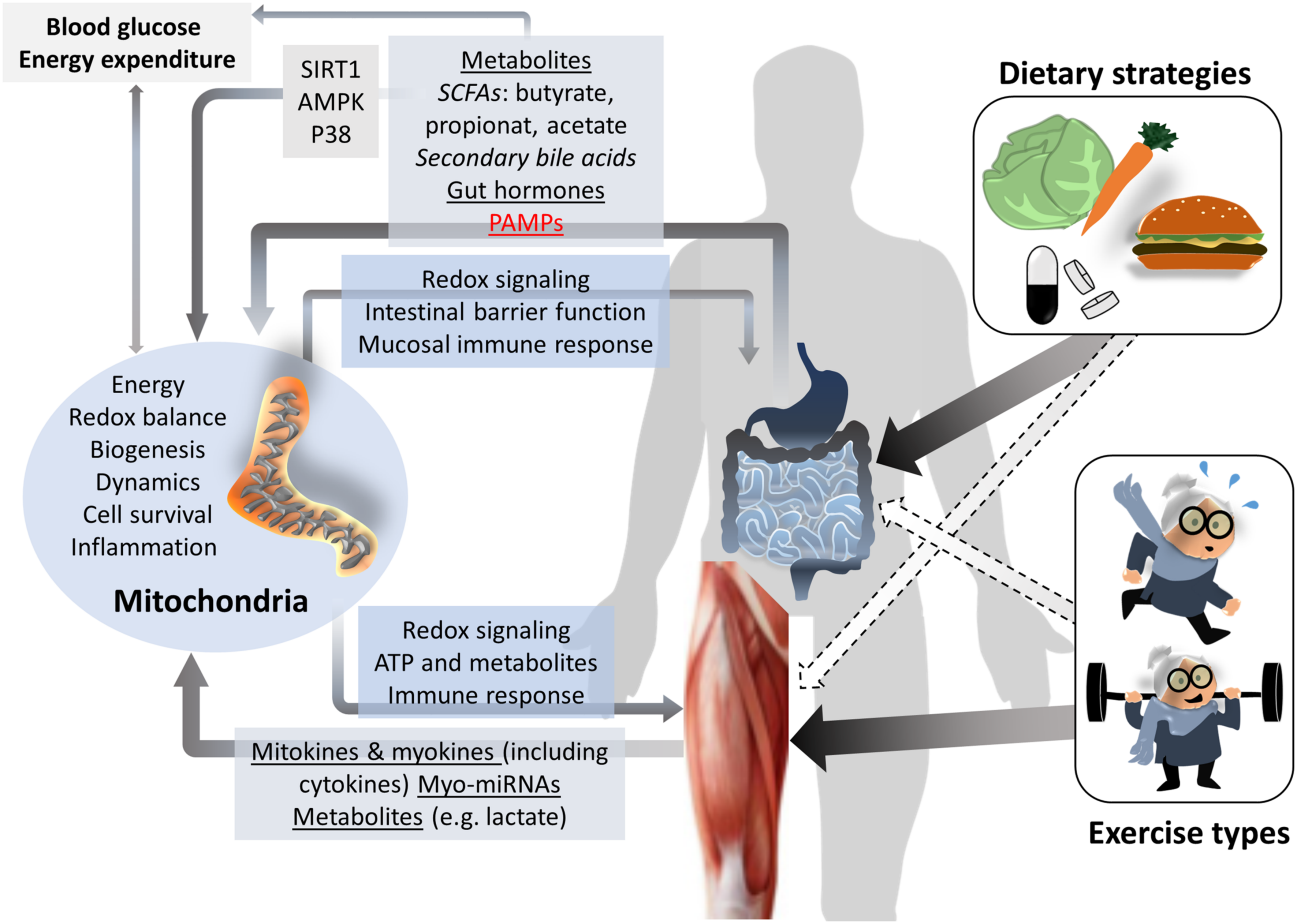
Potential mediation of the gut‐muscle axis by mitochondria. Metabolites and pathogen associated molecular patterns (PAMPs) are released by gut microbiota in response to dietary input and impact on host metabolism and mitochondria. Binding of short chain fatty acids (SCFAs) to G‐protein‐receptor coupled receptors on enteroendocrine L‐cells results in the secretion of metabolism‐modulating gut hormones. SCFAs also control mitochondrial biogenesis and ATP‐production and fatty acid oxidation via sirtuin 1 (SIRT1), AMP‐activated protein kinase (AMPK) and p38. Exercise induces the release of a variety of signalling molecules, including myo‐/mitokines, myo‐micro RNAs (myo‐miRNAs), many of which modulate mitochondrial functions. Mitochondria in turn regulate skeletal muscle and gut functions.

In summary, although it is likely that mitochondria are involved in the interplay of exercise, skeletal muscle, and gut microbiota (*Figure* 1), more research is required to elucidate the multidirectional signalling between different tissues, mitochondrial populations (i.e. in the skeletal muscle and in gut tissues), and microbiota. Furthermore, growing evidence supports the notion that different exercise modalities (such as type, duration, frequency, and intensity) elicit differential benefits for muscle mitochondria.^40^ It remains to be investigated, whether specific exercise recommendations to prevent age‐related sarcopenia differ in their effects on muscle (and potentially gut) mitochondria. In one recent study, HIIT increased markers of mitochondrial biogenesis, mitochondrial fusion, and mitophagy in obese older adults (Gouspillou *et al*. JCSM, in press) and acted synergistically with protein ingestion and L‐citrulline supplementation for increasing myocellular protein synthesis, muscle hypertrophy and strength.^46^ Based on these promising results more comparative studies on the efficiency, mode of action and mediating role of mitochondria of different exercise regimes in combination with relevant dietary strategies are required.

## Dietary measures to support resistance training and gut microbiota

Although a systematic review does not support important benefits of dietary supplementation combined with exercise training in the prevention and treatment of sarcopenia in subjects aged 65 or older,^47^ sophisticated nutritional strategies are expected to promote protein biosynthesis, growth, and/or maintenance of skeletal muscle^48^ as well as favourable effects on gut microbiota.^21^ While benefits of various dietary strategies—including protein, essential amino acid, polyunsaturated fatty acids, and antioxidant supplementation—on muscle mass and function in healthy elderly are widely accepted, the effects for frail populations are less clear.^2^

An increase in protein intake is generally considered the cornerstone dietary measure for preventing and treating age‐related sarcopenia, in association with exercise.^49^ However, a shift towards high‐protein diets can be associated with alterations of gut microbiota composition, and reduction of synthesis of important mediators of the gut‐muscle axis, such as SCFAs.^50^, ^51^ Such changes are exacerbated by a specifically increased ingestion of proteins of animal, and not vegetal, origin.^51^ Notably, in contrast to moderate protein supplementation, a very high protein intake is not advantageous for muscle strength enhancement during RT and this is likely due to its effects on gut microbiome function.^52^

Thus, microbiome‐centred dietary strategies to counteract age‐related sarcopenia should include not only moderate amino acid and protein supplementation but also balanced levels of polyunsaturated fatty acids, fibres, and antioxidants.^20^ Recent data from the US National Health and Nutrition Examination Survey suggest that increasing dietary fibre intake towards recommended levels (∼28–34 g/day) is associated with improvements in muscle mass and strength in adults aged 40 years and older.^53^ Plant and fibre‐rich dietary choices are in fact associated with a more diverse and compositionally distinct microbiota and with a greater potential to produce SCFAs.^54^ The Mediterranean‐style diet fulfils these criteria and can indeed induce positive changes in gut microbiota composition and function that are associated with reduced frailty and improved physical performance.^55^

## Conclusions and future perspectives

The gut microbiota has emerged as a powerful modulator of musculoskeletal health and disease and exercise likely is an important mediator. Exercise is associated with increased microbiota biodiversity and favours, for example, butyrate‐producing taxa with beneficial metabolic functions, which may contribute to the benefits of regular physical activity on human health. Effective strategies aimed at counteracting age‐related sarcopenia should consider the effects of exercise and nutrition on the gut microbiota. RT and balanced dietary intake of proteins and fibres are the interventions with the highest potential of inducing favourable changes in the gut microbiota, through mediation of microbial metabolites including SCFAs that have a known modulatory effect on muscle anabolism and chronic inflammation.

Exercise training also improves muscle mitochondria functions, which in turn regulate skeletal muscle and possibly gut functions. Mechanistically, mitochondria likely are key players in exercise–microbiota–muscle interactions. They are essential in skeletal muscle function during and following exercise and both regulate and are regulated by the gut microbiome. How mitochondrial populations (e.g. in the gut and the muscle) communicate and which effects physical activity and exercise exert on the reciprocal interactions of gut mitochondria and microbiota requires more research.

In conclusion, dietary strategies have the potential to support exercise‐induced adaptations and prevent age‐related microbiota dysbiosis and thus may be effective against age‐related sarcopenia. However, the—potentially synergistic—interaction between dietary interventions and exercise programmes against sarcopenia is insufficiently understood, especially from a clinical point of view.^56^ Traditionally, the investigation of dietary and exercise strategies on aging‐related factors in humans is complex and outcomes are determined by individual predispositions (genetic make‐up, general health status, dietary and physical activity habits, etc.) and variations to exercise adaptations and dietary interventions. Differences in microbiota likely are among these determining factors. Thus, future trials investigating the combination of nutritional and exercise interventions against age‐related sarcopenia should consider also gut microbiota composition and function among their endpoints, to disentangle the complex mechanisms of the gut‐muscle axis.

## Conflict of interest

The authors declare no conflict of interest.

## Funding

This work was financially supported by grants from the Austrian Federal Ministry of Education, Science and Research represented by the Austrian Research Promotion Agency (FFG) as part of the ERA‐Net Cofund HDHL‐INTIMIC (grant number BW000017276).
